# Equal North: how can we reduce health inequalities in the North of England? A prioritization exercise with researchers, policymakers and practitioners

**DOI:** 10.1093/pubmed/fdy170

**Published:** 2018-10-20

**Authors:** M Addison, E Kaner, P Johnstone, F Hillier-Brown, S Moffatt, S Russell, B Barr, P Holland, S Salway, M Whitehead, C Bambra

**Affiliations:** 1 Institute of Health and Society, Newcastle University, Baddiley Clark Building, Richardson Road, Newcastle Upon Tyne NE2 4AX, UK; 2 Public Health England, North of England, Blenheim House, West One, Leeds LS1 4PL, UK; 3 Department of Sport and Exercise, Durham University, 42 Old Elvet, Durham DH1 3HN, UK; 4 Institute of Psychology, Health and Society, Department of Public Health and Policy, University of Liverpool, Whelan Building, The Quadrangle, Liverpool L69 3GB, UK; 5 Lecturer in Public Health, Division of Health Research, Faculty of Health and Medicine, Lancaster University, Furness Building, Lancaster LA1 4YG, UK; 6 Department of Sociological Studies, The University of Sheffield, Elmfield, Northumberland Road, Sheffield S10 2TU, UK

**Keywords:** Delphi, engagement, equity, health inequality, social determinants, social policy

## Abstract

**Background:**

The Equal North network was developed to take forward the implications of the Due North report of the Independent Inquiry into Health Equity. The aim of this exercise was to identify how to reduce health inequalities in the north of England.

**Methods:**

Workshops (15 groups) and a Delphi survey (3 rounds, 368 members) were used to consult expert opinion and achieve consensus. Round 1 answered open questions around priorities for action; Round 2 used a 5-point Likert scale to rate items; Round 3 responses were re-rated alongside a median response to each item. In total, 10 workshops were conducted after the Delphi survey to triangulate the data.

**Results:**

In Round 1, responses from 253 participants generated 39 items used in Round 2 (rated by 144 participants). Results from Round 3 (76 participants) indicate that poverty/implications of austerity (4.87 m, IQR 0) remained the priority issue, with long-term unemployment (4.8 m, IQR 0) and mental health (4.7 m, IQR 1) second and third priorities. Workshop 3 did not diverge from findings in Round 1.

**Conclusions:**

Practice professionals and academics agreed that reducing health inequalities in the North of England requires prioritizing research that tackles structural determinants concerning poverty, the implications of austerity measures and unemployment.

## Background

The North of England (The North of England is defined geographically as the North East, North West and Yorkshire and Humberside.) has persistently poorer health than the rest of England and the gap has widened over 4 decades and five governments.^1,2^ Since 1965, this equates to 1.5 million excess premature deaths in the North compared with the rest of the country.^3^ Life expectancy is 2 years less for both men and women in the North compared to the South, mirrored by substantially higher rates of premature deaths from cancer and cardio-vascular disease (Table 1). Whilst the North represents 30% of the population of England it includes 50% of the poorest neighbourhoods,^1^ and tends to have worse health than places with similar levels of poverty in the rest of England.^1,2,4,5^ There is also a steeper social gradient in health within the North than in the rest of England.^6^

**Table 1:** Key health outcomes by English region, 2015^1^ (reproduced with permission from author and Policy Press)

	Population (millions)	Life expectancy at birth (LE, years)		CVD deaths (<75 years /100,000)	Cancer deaths (<75 years /100,000)	Diabetes % (>17 years)	% Obese or overweight (>16 years)
		Men	Women				
**NORTH** ^a^	15	78	81.9	89.6	161.4	6.5	66.5
North East	2.6	78	81.7	88.8	169.5	6.5	68.0
North West	7.1	78	81.8	92.8	159.8	6.5	66.0
Yorkshire and Humber	5.3	78.5	82.2	87.3	155.0	6.4	65.4
**SOUTH** ^b^	38	79.8	83.6	74.3	138.7	6.2	63.3
East Midlands	4.5	79.3	83.0	80.0	143.8	6.6	65.6
West Midlands	5.6	78.8	82.8	82.1	147.8	7.1	65.7
East of England	5.8	80.3	83.8	70.0	136.0	6.0	65.1
South West	5.3	80.1	83.8	80.1	136.5	6.0	57.3
London	8.2	80	84.1	66.4	134.0	5.6	63.1
South East	8.6	80.4	83.9	67.1	134.3	5.9	62.7
**ENGLAND**	53	79.4	83.1	78.2	144.4	6.2	63.8

^a^Author calculated mean of NE, NW, YH; ^b^author calculated mean of EE, EM, L, WM, SE, SW.

The causes of these spatial and socio-economic health inequalities are complicated and contested—both in research and policy terms in England and in other high-income countries. Factors include: (i) unequal social and spatial distribution of behavioural risk factors—including smoking—as a result of adverse responses to the external world, (ii) income and other material factors such as access to goods and services and exposures to physical risk factors, (iii) psychosocial factors such as domination/subordination or powerlessness—and the effects of the biological consequences of these feelings on health, (iv) an accumulation of different types of disadvantage over the life course and (v) political and economic structures such as the welfare system.^7^

These varied ways of locating the causes of inequality have distinct implications for what should be done to reduce health inequalities particularly in terms of whether interventions should focus downstream (on individuals and their behaviour or psychosocial resilience), upstream (such as interventions to improve the redistribution of income and life chances) or some combination of action at multiple levels. Much of public health policy in England^8^ and elsewhere has favoured downstream, behavioural approaches. However, there is increasing awareness, especially amongst the public health community, that these might actually increase health inequalities. Identified as intervention generated inequalities, these can result in benefiting less disadvantaged groups.^9^ Upstream approaches focusing on the social determinants of health operating within a complex system might be more effective.^10–12^

In 2014, Public Health England commissioned the Independent Inquiry into Health Equity for the North of England to explore the extent and causes of the North South health divide and health inequalities within the North. The resulting ‘Due North’ report^6^ made four sets of recommendations, to: (i) tackle poverty and economic inequality within the North and between the North and the rest of England; (ii) promote healthy development in early childhood; (iii) share power over resources and increase public influence on how resources are used to improve the determinants of health; and (iv) strengthen the health sector’s role in promoting health equity. It also made various research recommendations and in response to these, Public Health England North set up the Equal North network in partnership with Fuse (the Centre for Translational Research in Public Health), LiLaC (Liverpool and Lancaster universities collaboration for Public Health Research), the University of Sheffield and the NIHR School for Public Health Research (SPHR). Equal North is a research network of academics, policy and practice members. Its aim is to follow up the Due North research recommendation to identify areas of priority for local agencies in terms of reducing health inequalities. The network currently has over 500 members who were invited to join via events, email distribution lists and social media. Upon joining the network members indicated their area(s) of interest around health inequalities, which as a whole were very heterogeneous.

The aim of this study is to understand what the key priorities are for action and how research can best address these to reduce health inequalities’ by utilizing a prioritization and consensus building exercise amongst Equal North members.^13,14^

## Methods

**Fig. 1:**
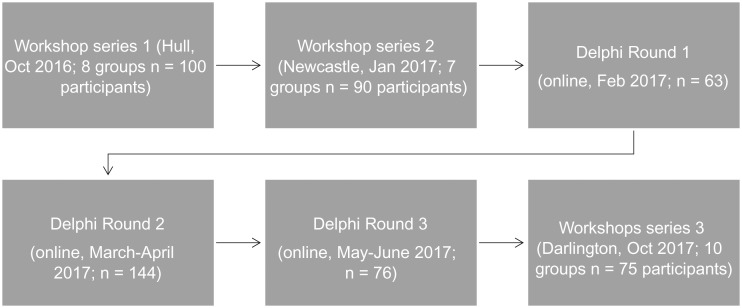
Methods: Flow through study of workshops and Delphi survey.

Study participants were the 368 registered members of the Equal North Research and Practice Network up to May 2017: 46% practitioners, 54% academics; 73% female; 38% from the North East, 35% Yorkshire and Humber, 21% from the North West and 6% are not regionally based. Members had an opportunity to contribute (Fig. 1) via a mixed methods approach. There were three rounds to the Delphi Exercise: Round 1—item generation facilitated by Workshops 1 and 2, as well as an online survey; Round 2—ranking of items via online survey, and Round 3—re-rating after median group result is known via online survey. Workshop 3 took place once all rounds were completed and enabled triangulation of results. All data is anonymised. All non-responders to the survey were followed up with two reminder emails in each round.

### Round 1: Workshops 1 and 2, and online survey

Workshop participants comprised 190 researchers, policymakers/practitioners working in public health attending three general inequalities events (only the workshop was focused on the study aim). At each workshop face-to-face interactive groups broadly scoped key issues prior to the Delphi to inform the design of the survey (Workshop 1, eight groups, *n* = 100 participants, 30 min/25 min discussion; Workshop 2, seven groups *n* = 90 participants, 60 min/55 min discussion). Group sizes ranged from 4 to 12 people and were all structured around facilitated discussion (conducted by one facilitator, one scribe) and a short scoping and priority exercise. No presentations were given at the beginning of the workshops, specifically, group participants were asked to discuss and generate lists for the following questions

- What causes inequality in the North and the North–South divide?
- What are the key inequalities in the North?
- What needs to be done locally and regionally to reduce inequalities in the north?

Participants then rated all items in terms of ‘urgent and important’, ‘not urgent but important’, ‘urgent but not important’ and ‘not urgent and not important’ for research.

Participation was entirely voluntary. Participants were made aware that discussion, whilst not audio-recorded, would inform on-going analysis around research priorities and Round 1 of the Delphi online survey. Anonymised notes were taken by an assistant in each group.

The online Delphi survey sought opinions on how best to tackle health and social inequality across the north of England and to identify future research priorities. Round 1 of the Delphi online survey aimed to generate ideas about priorities for tackling health inequalities and consisted of five open-ended questions (see Table 2), taking 10 min to complete online. All 368 members of the network were invited by email to complete the survey, and 63 (17%) did. Responses were combined with data collected from earlier Workshops 1 and 2.

**Table 2:** Round 1 key item generation from Workshops 1 and 2, and Round 1 Delphi Survey

Key questions: 1.What are the top three health inequalities issues in the north? 2. What are the top three health inequalities issues in your local area? 3. What evidence gaps are there that need filling?		
Overarching themes	Linked issues	Evidence gaps
Infrastructure	Roads Poor transport links Access/affordability	Value of joined up, inter-sectoral approaches—PH and voluntary sector Asset-based interventions rather than deficit or mitigation approaches Developing and evaluating proportionate universalism interventions Impact of devolution
Poverty/deprivation	Low wages Working poor Welfare cuts Food Banks Shame/Stigma Gambling and Debt	Effectiveness of new financial models/policies Economic evaluation of inequality reduction interventions—cost-effectiveness, wtp, E-B allocation, impact of cuts
(Un)Employment	Paucity of jobs Educational requirements	Identifying specific links between decision-making about jobs, economy and health outcomes
Education	Early years School readiness Lack of good quality teachers	
Housing and planning	Unhealthy/unfit housing Lack of affordable homes Lack of Accessible homes Homelessness	
Environment	Rural Isolation Access to green space ‘Broken windows’	Effectiveness of local actions, community control, community-led (priorities for action) Barriers/facilitators to community engagement/participation
Substance misuse/smoking	Alcohol Legal highs and illicit drug use Smoking	Interventions to address new/emerging health challenges
Chronic illness	Aging population in The North CVD, Respiratory Co-morbidity	
Obesity/childhood obesity	Diet/affordability of and access to (healthy) food Educational impact on health Physical activity	
Early years	Education Early interventions Access to healthy foods Breastfeeding	Effectiveness of family based interventions at reducing health/social inequalities
Mortality/life expectancy	Higher rates of chronic illness (e.g CVD, respiratory Unhealthy behaviours (e.g smoking, substance misuse)) Pockets of high socio-economic deprivation	Interventions to achieve healthy life expectancy—longer term effects of interventions
Mental health	Access to services Impact of poverty / deprivation	Effectiveness of targeted mental health prevention
Social isolation	From wider society Within ‘communities’, rural settings Aging population	Interventions to reduce loneliness, isolation, social exclusion How best to support/enable key groups—long term: conditions, disabilities, unemployed, NEETS
Disability	Higher rates in the North Loss of services/implications of austerity/welfare cuts Access	
Poverty/Absence of aspiration	Learned help/hopelessness Lack of opportunities Nihilism and apathy Disconnected Youth Stigma Shame	
Opportunity	Lack of opportunities Lack of assistance in accessing opportunities Resource drain—mass exodus of talent pool	
Health lit. (and education)	Low health literacy Educational impact on health Low understanding of the healthcare system	How best to get evidence into practice (implementation)—key groups, current constrained environ
(Sub)culture/embedded behaviours	Unhealthy learned behaviours Socio-cultural reinforcement of problematic behaviours Unhealthy/fatalistic coping behaviours	Critical appraisal of Public Health research—re-balance structural drivers and lifestyle (drift) work

What is the key role of PH researchers in helping local policymakers and practitioners?* (Only asked in online survey Round 1)
Presenting/disseminating evidence—what works (intervention effectiveness and evidence syntheses) Generating high quality evidence of effectiveness (and implementation effectiveness) Collaborating to promote knowledge translation, knowledge exchange Working more closely with decision-makers, HWBs, local groups to understand local issues Working rapidly to provide timely evidence—even if this requires reducing methodological purity Collaborating to co-produce evidence (relevant, local) and owned by all parties Providing training/learning opportunities so policymakers have better skills to understand evidence Building multi-sector teams to help produce joined up evidence generation/interpretation Producing ‘how to guides’ so that local practitioners can generate evidence themselves Developing a handbook for local elected members on ‘their role’ in tackling inequalities Developing new methods, e.g. so social value can be measured as well as health outcomes Working at a higher scale, i.e. natural experiments and system changes Lobbying for effective change—based on their knowledge of current evidence of what works Developing (jointly funded) embedded researchers (conversely academic homes/bases for others) Conducting pragmatic, real world research work—focused on the North (i.e. not UK, international) Carrying out more health economics research—return on investment approach Becoming local community advocates rather than bystanders/observers

### Round 2: rating items in online survey

Round 2 was an online survey where all members of the network were invited to rate the 39 generated items, which emerged from earlier thematic analysis, via Likert scales, and 144 members did (39% of membership).

### Round 3: re-rating items in online survey

In Round 3, the 144 participants from Round 2 were then provided with a summary of the group median responses and invited to re-rate the 39 items (April–May 2017) (see Table 3). In total, 76 of the Round 2 participants did (representing 21% of the total Network membership).

**Table 3:** Round 2—rating priority items, key research questions and key role of public health researcher

Round 2: Q.1 To what extent do you feel the following issues are priorities for action in tackling inequalities across the North of England?						
	Definitely not an important priority (1) and not a very important priority (2) (%)	Neutral (3) (%)	Very important priority (4) and extremely important priority (5) (%)	Mean	Median response	Count
Historical legacy, investment, infrastructure, transport, entrenched health disparities	8.5	14.3	77.1	3.94	4	140
Poverty/austerity, income growth/financial exclusion, access to services	0.7	2.1	97.2	4.61	5	142
Unemployment, jobs, worklessness, fair wages, low pay	0.7	10.6	88.7	4.42	5	142
Education and skills, functional literacy/numeracy, health literacy	2.8	15.4	81.9	4.15	4	143
Communication, insufficient partnerships, current structures, poor systems	11.3	35.9	52.8	3.58	4	142
Democratic deficit, representation, accountability, having a voice	7	27.1	66	3.76	4	144
Environmental, pollution, climate change, air quality, respiratory	8.5	27.7	63.8	3.77	4	141
Long term conditions, mortality/life expectancy and later life/aging	6.4	17.7	75.9	4	4	141
Homelessness and housing	3.6	15	81.5	4.15	4	140
Child specific issues, child poverty, early life, immunizations, adolescence, breastfeeding	4.9	9.1	86	4.29	5	143
Discrimination, minority, key under-served groups	6.4	15	78.6	4.06	4	140
Mental health, hopelessness, limited networks	1.4	5	93.6	4.45	5	141
Obesity/diet and physical activity	9.8	24.5	65.8	3.75	4	143
Smoking and electronic cigarettes/vaping	16.8	34.3	49	3.36	3	143
Substance (mis)use, alcohol, drug use	11.2	23.9	64.8	3.63	4	142
						144

Round 2: Q. 2. To what extent do you think the following research questions should be addressed in the next 1–2 years?						
	Strongly disagree (1) and disagree (2) (%)	Neutral (3) (%)	Agree (4) and strongly agree (5) (%)	Mean	Median response	count
How effective are family based interventions at reducing health/social inequalities?	13.7	25.2	61.1	3.6	4	139
How effective are targeted mental health prevention interventions?	7.2	20.9	71.9	3.91	4	139
How can evidence be effectively put into practice (implementation)?	8	16.7	75.4	4.02	4	138
How effective are approaches to address/change social determinants of health/inequalities?	2.2	10.1	87.7	4.39	5	139
How effective are new financial models/policies including the implications of devolution?	9.5	28.5	62	3.74	4	137
How effective are local actions and community-led initiatives, and what are the barriers and facilitators to community engagement and participation?	6.5	14.6	78.8	4.11	4	138
How can specific and marginalized groups best be supported and enabled?	5	20.3	74.6	3.99	4	138
What is the cost effectiveness of inequality reduction interventions?	8.8	27.7	63.5	3.78	4	138
What is the value of joined up, inter-sectoral approaches?	9.5	35	55.5	3.65	4	137
Is there evidence to support asset-based, as opposed to deficit or mitigation based, interventions?	10.8	36.2	52.9	3.52	4	138
How can we develop and evaluate proportionate universalism interventions?	10.2	32.8	56.9	3.66	4	137
How can we develop and evaluate interventions to reduce loneliness, isolation, social exclusion?	4.3	12.3	83.3	4.17	4	138
						140

Round 2: Q. 3. What is the key role of public health researchers in helping local policymakers and practitioners?						
	Strongly disagree (1) and disagree (2) (%)	Neutral (3)	Agree (4) and strongly agree (5) (%)	Mean	Median response	Count
Collaborating across multi-sector teams to co-produce evidence that promotes knowledge translation, knowledge exchange.	1.40	10.90	87.70	4.32	4.5	139
Becoming local community advocates rather than bystanders/observers.	10.10	22.50	67.40	3.86	4	138
Lobbying for effective change.	4.30	20.30	75.40	4.04	4	138
Developing jointly funded embedded researchers and practitioners (e.g. secondment) and providing training/learning opportunities for policymakers and researchers.	2.90	15.90	81.10	4.17	4	138
Disseminating evidence on what works (e.g. intervention effectiveness and evidence syntheses).	1.40	10.10	88.40	4.35	4	138
Generating high quality evidence of effectiveness and implementation effectiveness.	2.20	10.20	87.60	4.34	5	137
Working rapidly to provide timely evidence	10.20	20.40	69.40	3.88	4	137
Producing ‘how to guides’ so that local practitioners can generate evidence themselves.	9.50	26.30	64.30	3.76	4	137
Developing a handbook for local elected members on ‘their role’ in tackling inequalities.	13.80	36.20	50.00	3.51	3.5	138
Shifting research and policy focus from the individual to structural causes of health/social inequalities.	2.10	10.10	87.60	4.39	5	138
Conducting pragmatic, real world research work, e.g. natural experiments—focused on the north.	2.90	7.30	89.80	4.36	5	137
Carrying out more health economics research (return on investment approach).	9.40	30.40	60.10	3.65	4	138

### Triangulation

Workshop 3 followed the same format as 1 and 2 and comprised 10 groups *n* = 75 participants, 45 min/40 min discussion, and took place after the Delphi survey closed, to triangulate the data.

## Analysis

Data generated from Workshops 1 and 2, and Round 1 online Delphi survey, were thematically analysed by the research team; similar issues were grouped together and discrepant ideas were retained, creating 39 unique items responses to Rounds 2 and 3 were entered into SPSS and analysed descriptively to produce medians, standard deviation and an inter-quartile range (IQR). Results indicated areas of priority, and an IQR of ≤1 highlighted key areas of consensus across the expert group (0 = high consensus).

## Results

### Round 1: Workshops 1 and 2, and online survey

The wide-ranging issues that were generated from Workshops 1 and 2, and Round 1 of the Delphi survey, are outlined in Table 2. In total, 253 individuals participated in item generation work (*n* = 190 participants from Workshops 1 and 2; *n* = 63 responses to survey). The response rate to Round 1 of the survey was 17%. The issues considered most urgent for research, policy and practice were linked to poverty and deprivation in the region and the impact on the more disadvantaged sections of the population. There was some discussion around how to translate evidence into practice in a timely way for more immediate impact on the determinants of health inequalities. It was recognized that this was complicated further due to local government budget constraints and a tendency for organizations across the public and voluntary sector to work in silos. Further, some participants (who were service providers) also reported that it was important to lobby local politicians around key priority issues in order to instigate change.

Overall, key overarching issues emerging from workshops and Round 1 of the survey tended to focus on the structural determinants of health inequality, these included issues around: unemployment and paucity of stable jobs; child specific issues linked to opportunity and ‘aspiration’; as well as poor mental health linked to isolation and feelings of stress related to poverty. Some participants within workshop groups steered discussion towards a focus on individualized behaviours that were harmful to health, such as substance and alcohol use, and unhealthy food choices, as well as issues around an absence of aspiration and a perception of worklessness entrenched amongst certain communities in the North. The majority of participants from Workshops 1 and 2 and the survey reported that research should be focussed on exploring ways to impact on structural inequalities in the different northern regions, and to understand what makes some communities able to withstand the impact of austerity measures. All participants from the network were asked to rate these items in Round 2 of the Delphi.

### Round 2: rating items in online survey

In Round 2, 144 participants responded to the survey (39%: out of a possible 368). Of these, 47% were practitioners and 53% academic.

### Round 3: re-rating items in online survey

In Round 3, 76 participants from the previous round responded (half of the Round 2 participants, giving a response rate of 21% of the total network membership, and of these half were practitioners). It was clear from some open-ended responses that a number of participants consulted with their respective teams and represented the views of their wider practice organization, indicating that findings may capture more views than the percentage reported.

### Consensus and divergence in Rounds 2 and 3

The findings from Rounds 2 and 3 (Tables 3 and 4) of the Delphi survey remained consistently focused, showing that the top priority for research, rated extremely important/important (4 or 5) by members, and with high consensus (IQR 0, 0.34 SD), should focus on issues of poverty and the implications of austerity, as well as the challenges presented through financial exclusion and uneven access to services (e.g. GPs, drug and alcohol, training). Whilst all academics rated poverty and the impact of austerity as the top priority in Rounds 2 and 3, the majority of practitioners in Round 2 signalled mental health issues to be a greater priority (Tables 3 and 4). Although mental health was consistently rated as a very important or extremely important priority by everyone, it was overtaken in Round 3 with a strong consensus (IQR 0, 0.528 SD) that members wanted unemployment and worklessness to be visible and developed as a research priority for the North (IQR 0, 0.46 SD). Child specific issues related to poverty, early life and adolescence increased in priority, with 93% of participants in Round 3 rating it as very important or extremely important. This was closely followed by issues related to education, skills and literacy with a median value of 4 (‘very important’).

**Table 4:** Round 2 and 3—top priority issues and questions for research

Issues for research	Round 2 (*n* = 144)						Round 3 (*n* = 76)					
	Total % rating either extremely [5] or very important [4] priority	*N* = Academics (72), Practitioners (62), *n* = 10 missing data. Rating either extremely [5] or very important [4] priority (*n* = )	Mean	IQR	SD	Median	% Rating either extremely [5] or very important [4] priority	*N*= Academics (35), Practitioners (35), *n* = 6 missing data. Rating either extremely [5] or very important [4] priority	Mean	IQR	SD	Median
Poverty/austerity, income growth/financial exclusion, access to services	96%	72, 58	4.61	1	0.569	5	100%	35, 35	4.87	0	0.34	5
Mental health, hopelessness, limited networks	92%	66, 60	4.45	1	0.659	5	97.3%	34, 33	4.7	1	0.528	5
Unemployment, jobs, worklessness, fair wages, low pay	88%	67, 51	4.42	1	0.708	5	98.7%	34, 35	4.8	0	0.46	5
Child specific issues, child poverty, early life, immunizations, adolescence, breastfeeding	85%	61, 55	4.29	1	0.903	5	93.4%	33, 32	4.6	1	0.76	5
Education and skills, functional literacy/numeracy, health literacy	81%	54, 55	4.15	1	0.781	4	92.1%	30, 34	4.3	1	0.749	4

Priority research questions	Round 2						Round 3					
	Total (*n* = 144) Rating either strongly agree [5] or agree [4]	*N*=Academics (72), Practitioners (62), *n* = 10 missing data. Rating either extremely [5] or very important [4] priority (*n *= )	Mean	IQR	SD	Median	Total (*n* = 76) Rating either strongly agree [5] or agree [4]	*N*=Academics (35), Practitioners (35), *n* = 6 missing data. Rating either extremely [5] or very important [4] priority	Mean	IQR	SD	Median
1. How effective are approaches to address/change social determinants of health/inequalities?	87.7%	59, 55	4.39	1	0.757	5	86.1%	32, 28	4.38	1	1.01	5
2. How can we develop and evaluate interventions to reduce loneliness, isolation, social exclusion?	83.3%	62, 47	4.17	1	0.833	4	81.9%	31, 26	4.01	1	1.01	4
3. How effective are local actions and community-led initiatives, and what are the barriers and facilitators to community?	78.8%	55, 45	4.11	1	0.922	4	80.5%	29, 27	4.04	1	0.971	4

When asked which research question should be prioritized by the Equal North network, several options achieved consistently high rankings but members did not reach a strong consensus (IQR < 1) in Round 3 (Table 4). However, Round 3 shows that 86% of the sample stated that they either strongly agreed^5^ or agreed^4^ that examining the social determinants of health inequalities and effective ways to change these should be the priority for research. Both academic and practitioner members were generally in agreement. Further, 92% (4.56 m) said that the role of researchers in the future should be to shift research and policy focus from the individual to structural causes of health and social inequalities (Table 5).

**Table 5:** Rounds 2 and 3—key role of health researchers

Key role of public health researchers	Round 2						Round 3					
	Total (*n* = 144) Rating either strongly agree [5] or agree [4]	*N*=Academics (72), Practitioners (62), *n* = 10 missing data. Rating either extremely [5] or very important [4] priority (*n* = )	Mean	IQR	SD	Median	Total (*n* = 76) Rating either strongly agree [5] or agree [4]	*N*=Academics (35), Practitioners (35), *n* = 6 missing data. Rating either extremely [5] or very important [4] priority	Mean	IQR	SD	Median
1. Shifting research and policy focus from the individual to structural causes of health/social inequalities	87.6%	66, 49	4.39	1	0.787	5	91.7%	32, 33	4.56	1	0.868	5
2. Conducting pragmatic, real world research work focused on the North	89.4%	64, 52	4.36	1	0.775	5	91.6%	33, 31	4.46	1	0.8	5
3. Disseminating evidence on what works (e.g. intervention effectiveness and evidence syntheses)	88.4%	59, 55	4.35	1	0.78	4	86.1%	28, 32	4.26	1	0.822	4

### Workshop 3

Insights collected from Workshop 3 triangulated with the data collected from Workshops 1 and 2, and the issues arising out of the Delphi, with the exception that novel psychoactive substances and problem gambling were new issues raised by participants.

## Discussion

### Main finding of this study

The aim in this exercise was to understand what members of the Equal North research network identify as priorities for action and research in the north.^6^ Key findings show a strong consensus across both practice and academics to prioritize tackling embedded health inequalities complexly linked to structural determinants around poverty, and the implications of austerity and unemployment. The workshop discussions linked the causes and consequences of health inequalities to low wages, welfare cuts and a growing sub-section identified as the ‘working poor’ (in-work but perceived to be in poverty). Concern was raised in all workshops around how to tackle these issues with increasingly constrained budgets in the public and third sector and limited staff and material resources.

A spread of research priorities were identified by participants, and whilst several research questions were rated highly (86% in Round 3 prioritized examining the social determinants of health inequalities and effective ways to change these), none reached a definitive consensus. Despite the causes of health inequalities being a contested issue within workshop discussions, a strong focus on the structural determinants (social, political and economic) of health was important to participants when prioritizing areas for further research. This indicated a desired move away from current UK policy agendas^1,4,11,15^—which have focussed on behaviour change interventions administered at the level of the individual, with short-term goals (e.g. CHD, diabetes)—towards upstream factors impacting on long term health inequalities.^6,16^ Working together meant that public health researchers were positioned as advocates for social change. Finally, future research should give due consideration to how the design and implementation of policy may lead to intervention generated inequalities.^9^

### What is already known on this topic

We know that inequality impacts on health resulting in reduced years in good health, reduced opportunities for improving life quality, lower life expectancy and increased poverty.^2,4,11,15,17,18^ The Due North Report^6^ identified that the main causes of health inequalities between the North and the South of England were differences in: poverty and power; exposure to health-damaging environments; prevalence of chronic disease and disability; and, opportunities to utilize positive and protective conditions for healthy lifestyles. Bambra’s^1^ in-depth exposition of the social, environmental, economic and political causes of health inequalities directs attention towards a more upstream agenda to shape policy and practice. The findings from this research exercise indicate that participants could identify both structural and individual determinants of health inequalities, but that their priority for research was to focus on upstream factors. This presents theoretical and practical challenges^19^ tackling health inequalities at both a micro and macro level to account for the complex impact on health.

### What this study adds

A breadth and depth of knowledge is contained with the Due North report,^6^ yet our exercise shows it is challenging to prioritize issues, share information and develop a joined up action plan^20^ across geographically disparate services, Clinical Commissioning Groups, Local Government and academic institutions. In particular, our study shows that participants want researchers to disseminate findings widely to policymakers and practitioners around best practice, case studies and the effectiveness of upstream interventions. It has provided a strong indication for the direction and priority for research questions, the level of interest amongst members, and the role of public health research that is specifically of concern to a northern cohort of academics, policymakers and practitioners.

### Limitations of this study

There was a low response to the online Delphi survey across the three rounds: 17% of network membership in R1, 39% in R2 and 21% in R3. This exercise was undertaken at a time when the network was expanding—hence, we used multiple methods of engagement and re-engagement. An additional question about role of research was added to Round 1 of the survey and was not posed to workshops. In Rounds 2 and 3 participants rated grouped options to question 1: participants may disagree with how these were grouped making ranking more difficult. There was a potential ceiling effect leading to high rankings of certain items although the IQR suggested consistent agreement and few outliers. Participants were self-selected with particular interests in health inequality and were also regarded as either a practice or academic expert and were not therefore a homogeneous group—although all worked in the applied public health field and had shared interests in how to reduce health inequalities. Delphi measures have previously been successfully used on mixed public health professional groups.^21^

## Conclusions

This research exercise highlights a strong consensus amongst practice professionals and academics that reducing health inequalities in the North of England requires prioritizing and tackling structural issues around poverty, the implications of austerity and unemployment. The highest rated area of research for policymakers and practitioners going forward is in areas that examine the social determinants of health inequalities and effective ways to change these. The Equal North network continues to grow, serving as a platform for information sharing, discussion and a repository of existing research and evidence.

## Supplementary Material

Equal_North_-_Health_Inequalities_reviewer_author_response_July_2018_FINAL_fdy170

## Relationship statement

The NIHR School for Public Health Research is a partnership between the Universities of Sheffield, Bristol, Exeter, Cambridge, UCL; The London School for Hygiene and Tropical Medicine; the LiLaC collaboration between the Universities of Liverpool and Lancaster and Fuse; The Centre for Translational Research in Public Health, a collaboration between Newcastle, Durham, Northumbria, Sunderland and Teesside Universities. The study was led by Fuse investigators at the University of Newcastle upon Tyne, UK.
