# Supplementary material for: Equal North: how can we reduce health inequalities in the North of England? A prioritization exercise with researchers, policymakers and practitioners
Source: J Public Health (Oxf). 2018 Oct 20;41(4):652–64. doi: 10.1093/pubmed/fdy170 (PMC6995035; doi:10.1093/pubmed/fdy170)
Supplement: Equal_North_-_Health_Inequalities_reviewer_author_response_July_2018_FINAL_fdy170 [file equal_north_-_health_inequalities_reviewer_author_response_july_2018_final_fdy170.docx]

**Equal North: How can we reduce health inequalities in the North of England? A prioritisation exercise with researchers, policymakers and practitioners**

Addison, M.^1^; Kaner, E.^1^; Johnstone, P. ^5^; Hillier-Brown, F. ^1^; Moffatt, S. ^1^; Russell, S. ^1^; Barr, B. ^2^; Holland, P. ^3^; Salway, S. ^4^; Whitehead, M^2^.; and Bambra, C.*^1^

Thank you for giving us the opportunity to respond to the reviewers’ detailed and incisive comments regarding our manuscript. We were pleased to see that reviewer 1 considered our priority setting exercise extremely useful, particularly for a public health audience relevant to the JPH. We were also encouraged to see that reviewer 2 considered this to be an important topic area, and that reviewer 1 thought that the north of England is a suitable case for prevention and could contribute towards best practice for other decision makers in other localities.

Please find our author comments detailed below.

*Reviewer / Author Matrix Response Table*

| **Reviewer 1 Response** | **Author Response** |
| --- | --- |
| 1. Workshops and Delphi processes are becoming increasingly popular forms of knowledge generation and yet I don’t think quality criteria or reporting standards for these types of methods are well established or agreed upon. What makes a good workshop and Delphi process? The current draft has a brief limitations section covering, for example, response rates. I’d like to see some information on the following for the workshops:    1.1. Length of the workshops, including length of time allocated for discussion.    1.2. Proportion attending more than one workshop    1.3. Were there any presentations prior to discussion? Researcher or practitioner led? Crucially, did presentations focus on or advocate social determinants of health strategies?    1.4. More detailed descriptions of attendees’ job positions (more than just academic vs practitioner) – ideally some indication of seniority and sector (public health, planning, education, etc). If you don’t have exact numbers then a more detailed text summary.    1.5. How was data recorded and by whom.    1.6. More details on how similar or different the formats of the 3 workshops were and what is meant by triangulation.    1.7. For the Delphi sessions, again a more detailed (numeric) break down of participants. Also (if known) the proportion who took part in different waves – or (if more appropriate) an explanation for why that isn’t important. | Thank you for these comments.  1.1 p.3 lines 25-27 have been amended to included workshop and discussion length  1.2 We are not able to provide this data because we did not collect identifiable data from workshop participants. As workshops were voluntary we would not expect persons to duplicate their participation in a workshop.  1.3. No there were no presentations prior to discussion in the workshops. This is clarified page 3, line 30  1.4 We do not have this data as we did not collect identifiable information from the participants in the workshop. Generic job sector was noted only - this was to facilitate anonymity.  1.5 pg 3 line 39 data was recorded by an assistant. No audio equipment was used and this was agreed by our ethics approval system.  1.6 All workshops used the same format – facilitated discussion with 3 principle questions– p3, line 28-29  1.7 methods section describes how participants who filled in the survey in round 2 were invited back in round 3 (n=76 took part in R3 out of a possible 144 people). |
| 2. The total number of workshop participants was roughly equal to the total number of survey participants. I’d like the authors to reflect more on that. The first two workshops are described as being intended ‘to inform the design of the survey’ and the manuscript’s Findings generally focus on survey data. This suggests that the survey was the main focus of the study, while the first 2 workshops were really a survey-design exercise; and the third workshop affirmed survey findings (whilst identifying two additional themes). If the authors think my summary is correct, then I guess they don’t need to revise their text – but if they think the workshops provided interesting data in their own right, rather than simply serve the survey, I would suggest revisions to bring this out further. | Thank you for your comments. Workshops 1 & 2 were intended to generate items to be used in the Delphi survey (p3, line 25). Item generation is a standard process when deploying a Delphi method in the first round. The third workshop was intended to check if any new items had been generated in the elapsed time, or if there were any discrepancies with findings from the 3 rounds. As the workshops were focused on structuring the items for the Delphi the text has not been amended in the manuscript. |
| 3. I got lost trying to read the multi-page tables at the end. Partly because of their size but also because they didn’t always map very well onto the study stages described in Figure 2, and because the headings weren’t always clear.    3.1. For example table 2 – the table doesn’t make it clear if it is based only on Delphi survey round 1, or if it is also based on the first 2 workshops. It refers to questions about health inequality issues, evidence gaps, and what research can do to help – but the table only describes issues.    3.2. Table 3 has inconsistent formatting (e.g. use of capitals on second page) that makes it unclear whether we are looking at one big or 3 smaller tables – and it would be helpful to make it clearer that all parts of that table refer to round 2 delphi (if that is the case). Similarly I found myself loosing track of what was being presented in the first second and third pages of table 4. Also, which of these tables is the really important bit? Does the Delphi process presume that the 3rd round is the one everything else is working towards?    3.3. Something needs to be done to make this all easier to follow. Here is a suggestion.         3.3.1. One table or panel summarising main themes / findings emerging from workshops 1 and 2 (and please clarify somewhere that these workshops were broadly similar in format, or allude to the differences).         3.3.2. One table in the main text showing a summary of whatever you think is the most important data from the various surveys.         3.3.3. Put the rest of the survey findings into an online supplemental table(s).         3.3.4. A final panel in the main text showing triangulated (and non-triangulated) findings emerging from workshop 3. | Thank you for your comments – we have labelled the tables more clearly.  3.1 This is now clarified p12. We have also added an additional column to table 2  3.2 Thank you this formatting issue has been corrected on p14-16. Table 3 presents the data collected in round 2 which is discussed p5 comparing changes in priority compared with round 3 when participants are presented with the group median response and an opportunity to change their answers. The Delphi process is a way of presenting consensus around key issues and the final round (3) presents the final results from the study.  3.3 Thank you for these suggestions. The journal guidelines limit the number of tables and figures we can include in the paper so we are constrained to follow the suggestion to add more tables. However, we can show that we are including the important tables in the main body of the paper:    Table 4 presents the important results i.e. top 5 issues across round 2 and 3 demonstrating strong consensus; this table is to be included in the results section on p5. Table 5 also presents the three top identified key roles for Health Researchers from round 2 and 3 and is to be included on p6.  Table 3 (which contains all of the data we collected for round 2 and 3) is to appear in the appendix at the end of the paper (indicated on p4). |
| 4. A devil’s advocate point: how can the authors convince readers that the findings were not skewed by the researchers’ own well established (e.g. see many of the publications referenced in this manuscript) interest in social determinants approaches? I imagine the answer lies in providing assurance that workshops did not include leading presentations, and by comparing the way practitioners and researchers answered the surveys. | Thank you for this comment. We have endeavoured to be completely transparent with the data we have collected which is why we include table 2 (Key item generation from workshops 1, 2 and round 1 of Delphi Survey) and Table 3 which includes all the results we collected from round 2 so the reader is able to clearly assess that findings are not skewed. We have also highlighted on p2 that no presentations were provided before the workshops. Round 1 of the Delphi survey also ensured that participants were able to complete the questions without undue influence. |

| **Reviewer 2 Response** | **Author Response** |
| --- | --- |
| 1. **Abstract** A. Page 1 Line 19   Need to state aim of research prioritisation exercise and also of paper.  b. Page 1 Line 20  The method is too short. Currently the information required is contained within the results section of the abstract. This needs to be put into the methods section.  1c. Page 1 Line 25  There is a mix between methods and results here. The results don’t give the results for round one. It is my understanding that thematic analysis of the workshops plus the open ended survey generated a list of 39 items used in round 2.  1d. Page 1 Line 26  Again this is method. Here we need the top items coming out of round 1.  1e. Page 1 Line 27  Replace method information with just the results. State what remained the top priorities and if there were any changes from round 2.  1f. Page 1 Line 27  If the workshops at the end of the study were part of the method, surely something should be said about what they found.  Page 1 Line 33  What about policymakers? | 1a. Page 1, Line 19 – amended  1b. Page 1, Line 20 – amended  1c. Line 25 – amended  1d. Line 26 – items have not been rated yet in round 1 so it is not possible to indicate top results here  1e. Line 27 – amended  1f. Line 33 – amended  1g. policymakers are included under ‘practice’ |
| 2. **Background**   2a. Page 2 Line 31  Intervention generated inequalities- a small bit of elaboration required with a supporting reference.  2b. Page 2 Line 35-36  This needs to be slightly reworded.  2c. Page 2 Line 41-42  Table 1 needs to be referred to in the text and also analysed and interpreted. It is a useful table in terms of setting the context of the study and should be included.  Page 2 Line 43-50  This overlaps with the first paragraph in the background section. Suggest incorporate into paragraph 1 and only have the North report findings here.  2d. Page 3 Line 11  Reword ‘take up’  2e. Page 3 Line 13  Although I do like maps, I feel that figure 1 should be removed as it doesn’t really add any value to the paper. The information is already within table 1 and there are no gender differences.  2f. Page 3 Line 15-20  This information about the network needs to be reduced as it doesn’t really add any value to the paper. Information in terms of how people were selected to join the network need to be included. This is very important in terms of using the Delphi technique.  2g. Page 3 Line 22  The aims and objectives of the study should be clearly stated. At the moment they are implicit and interwoven into the aims of the working group | Thank you for these comments  2a. Page 2, line 31 – amended and ref included  2b. 41- 42 amended  2c. amended  2d. Page 3 - Line 11 amended  2e. Page 3, line 13 – amended  2f. Page 3 lines 15-20 this has been added into the main body of the paper.  2g. Line 22 - clarified |
| 3. **Methods**   3a. The study methodology is presented in a way that is somewhat confusing to the reader. For example workshop numbers and round numbers. It is confusing in terms of who were the participants for each workshop and round.  3b. Were they taken from the same pool? I don’t think figure 1 adds much value or facilitates this issue. Consider removing or presenting a better figure. If the methodology was restructured it would help. The 2 workshops and the open ended survey essentially are the Delphi study round 1. Workshop 3 could possibly be repackaged as Delphi study round 4. It will flow much better if  presented in that way. This all depends on who the participants were. The structure should be reworked to make more user-friendly. The issue of whether the first two workshops should have been included in addition to the open ended questionnaire can be considered in the discussion. Page 3 line 26  More information on study participants is required for both workshops and surveys. Were the workshops only open to network participants. It is said that they were people working in public health.  3c.How were they invited to attend?  Was the conference specifically set up to address the study aims? What is the criteria for joining the network.  3d. The Delphi technique is designed to get consensus opinion among experts. What criteria are we using to define these people as experts. It appears that there are three different types of participants- researchers, policy makers, and practitioners. More detail for each of these categories would be helpful. Surely these participants would have expertise in different areas and not be from a homogeneous group. It may have been better to undertake the Delphi process on each group separately to compare and contrast priorities. This issue needs to be raised in the discussion.  3e. Page 3 line 36  Workshop 3 should be dealt with elsewhere as part of an improved structure. More information about the methodology employed for workshop 3 should be provided.  3f. Page 3 line 41-45  I am wondering why the role of research and research priorities wasn’t asked in the workshops, since this was a core aim of the study and of the working group.  3g. Page 4 line 7  Should this be table 2? Table 2 refers to results and should not be referred to here. Rather I would list the open ended questions.  3h. Page 4 line 7  What is the logic of having a different set of questions to the workshops?  3i Page 4 line 8  Are the 368 members of the network the same people that attended the workshop?  3j Page 4 line 8  Rephrase ‘did so’  3k. Page 4 line 11  What were they asked to rate the 39 items in terms of?  It appears from table 3 that respondents were also asked two additional questions. What is the rationale for the additional questions that are given in table 3? This needs to be included in the method. Page 4 line 27  What were they asked to re-rate the 39 items in terms of? | 3a. Thank you for these comments. We have added further clarification about the methods process at the beginning of this section. The methods have also been restructured as advised by the reviewer to make the different rounds of the Delphi clearer.  3b. Study participants were sampled from the Equal North Research Network (p4.line 31-34). Members were invited to join the network via email, events, flyers and social media (p. 4 line 20-21)  3c. The conferences were not set up solely to address the study aims. The workshops were embedded as part of a programme. This is clarified in this section.  3d. More detail has been added in the methods section, and heterogeneity has been noted in the limitations section. Previous use of Delphi methods amongst mixed groups of public health professionals (researchers, policy and practice) has also been referenced (Journal of Public Health, 2010, 32: 496–505.).  3e. Thank you for this it is now part of an improved structure. Please see page 5.  3f. Questions were generated for the workshops first. Participants were asked to prioritise their top 3 health inequalities issues in their local area and in north, as well as evidence gaps - generating items to be ranked and prioritised in round 2 and 3. An additional question about role of research was added to round 1 of the survey and this is made explicit in table 2. This is also added to the limitation section.    3g. This has been deleted.  3h. Pg4, line7  The questions for Round 1 workshops were developed first and after assessment of initial data collected an additional open question was added to the Delphi round 1 survey ‘The key role of PH researchers in helping policymakers and practitioners’. This data is added to table 2 and only refers to items collected from survey round 1.  3i Page 4 line 8  Not every member of the Equal North network attended a workshop, but everyone is included in the round 1 survey.  3j. Page 4 line 8  Deleted  3k. Page 4 line 11  Thank you for this. We can clarify that the 39 items were rated on a 5point Likert scale. The 39 items were in 3 sets, one set looked at establishing priority, the second set examined consensus regarding research questions, and the third set looked at the role of public health researchers |
| 4. **Results**   Page 4 line 46   a. This is round 1 really and the way this is structured is confusing. The heading says workshops yet the analysis is for the workshops and the open ended survey. Can the outputs from each be identified as it appears that all the information has been obtained from the workshops. b. Page 4 line 49   Table 2 isn’t user friendly and is very difficult to follow. Themes and issues are summarised too much, making them too broad and difficult to determine the specific issues that arose. Results should have been organised around the 4 sets of questions that were asked. This would provide the reader with a more in depth understanding of the issues that were raised and also provide a rationale for the questions that were used in subsequent phases of the research (e.g. table 3). From the table it appears that there were 18 key themes. Perhaps it would have been better just to state that there were 18 themes (perhaps listing them) and then giving an overview of the top 5 themes with a few quotes from participants to add some depth to the results.   c. Page 4 line 49-57   Are the issues referred to the top themes emerging from the analysis   d. Page 5 line 3-14   This analysis here sounds like it’s all from the workshops.   e. Page 5 line 9-14. This is method not results. Themes should be classified in terms of structural versus individual to help emphasise this point. f. Page 5 line 16   No analysis is given of workshop 3. Results need to be grouped by round and question areas asked. Page 5 line 18-19 Again structure of paper very confusing and it is unclear where the results of round one are presented.   g. Page 5 line 20-27   The findings of round 2 (table 3 q1, q2, and q3) should be analysed. Results should have been disaggregated by the three main respondent types.   h. For table 3 q1, it would be useful to be reminded of the question that was asked. i. Also, only 15 items are listed, yet 39 items were supposed to have been rated. Are these the top 15? Pleas clarify in the paper. j. For table 3 q1, It is difficult to interpret the rating of items because many are broad and somewhat ambiguous. In addition each scale item covers more than one issue, meaning that it is difficult to interpret what the respondent was rating (e.g. historical legacy, investment, infrastructure, transport, entrenched health disparities all in one scale). This is a significant weakness which at this point cannot be overcome. This has to be dealt with in the limitations section. k. Page 5 line 25   Half were practitioners, but also need to state how many were researchers and how many were policy makers.   l. Page 5 line 29-42   Analysis here should focus on the key changes between rounds 2 and 3 for the three questions that were asked. All analysis should compare between the three groupings (policy makers, practitioners, and researchers) and state whether there were statistically significant differences for the quantifiable results.   m. On page 17, the table should clearly label round 2 and round 3. n. Page 5 line 51-57. Results should have been classified in terms of structural and individual to support this analysis. This paragraph mainly refers to a comparison between the workshops and round 1 and should have been dealt with where round 1 results are presented (except the analysis of table 5). As stated earlier, the workshop and round 1 of the Delphi study essentially form round 1 of the Delphi study and should be presented in the way, as the current structure is confusing. The merits of employing this approach to round one can then be dealt with in the discussion. | 4a. Thank you for this. The results section has been restructured so that it follows the same formatting for the methods section (round 1/2/3).  4b. Page 4 line 29- The table presents consolidated issues from discussions in the workshops so that they can be used for the Delphi survey method to rank and prioritise items. An extra column has been added to make the data about evidence gaps clearer. It would not be appropriate to open out categories for the Delphi method. The workshops were not audio recorded so we are unable to provide quotations.  4.c Yes they were the main themes  4d. and 4e This analysis is not all from the workshop and this is made clearer in the text.  4f.Text regarding workshop 3 has been added. Results have been grouped by rounds as suggested.  4g. The results by respondent type would be too small to be regarded as meaningful. This is why we have used overall sample as key indicators. We have however made these data available in table 4.  4h. We have added the question back in to table 3 question 1.  4i. The 39 items have been grouped under question 1, 2 and 3.  4j. This has been acknowledged in the limitations section.  4k. We have stated at the beginning of the paper the proportions of the Equal North network that were categorised as practice and academics.  4l. This has a new subtitle: Consensus and divergence in rounds 2 and 3. We are not focusing on statistical significance between groups in this paper or through the Delphi method.  4m. The text has been enlarged to make this clearer  4n. This text has been integrated into the new format of round 1 /2 /3 suggested earlier in this section by the reviewer. Structural and Individual is framed in the discussion section. |
| 5. **Discussion**   Page 6, line 7-36   a. The main findings section of the discussion should be structured better. The key quantifiable findings should be given, elaborated upon with examples from the workshops and round one open ended survey, plus supporting literature, stating implications. How issues identified are different in the north compared to the rest of England needs to be given. Currently the value of the study is lost as the depth behind the key issues that are identified are lost.   5b. Page 6, line 23  “growing subset identified as the working poor.” This concept needs to be explained giving more depth.  5c. Page 6, line 24  “constrained budget and paucity of resources”- again more depth required.  5d. Page 6, line 25-30  Again the data in the results needs to be presented in a way that supports this statement. Also what were the most important structural determinants. Need literature to support the need for structural determinants. Also need to elaborate on fact that UK policy is focusing on behaviour change interventions.  5e. Page 6, line 33-37  I didn’t see how this linked with the findings.  5f. Page 7, line 3-7  I didn’t see this issue within the results.  5g. Page 7, line 14-20  Limitations section needs to be expanded. For example there is a need to comment on the merits of open-ended type on line surveys. It may have been better in terms of meaningful responses to have done 1-1 interviews on a smaller sample. Need to mention how workshops were combined with open ended surveys and the merit of that. Also having three different types of expert would not as far as I am aware be recommended for the Delphi technique. This issue needs to be discussed.  5h. Page 7, line 18-19  Need to expand ceiling effect point. | 5a. The discussion section is structured according to the journal guidelines which specific what is to go into each subheading. We can therefore not restructure this section. However, additional references have been added as suggested. The main findings from the study are outlined under ‘main findings of this study’. Word count limits outlining the specific results again here. We are unable to compare the results to the rest of England specifically as the exercise focused on the north.  5b. additional text added  5c. additional text added  5d. This section presents what is already known on this topic in available literature. The most important structural determinants are outlined in the paragraph above ‘main findings’  5e. additional text added  5f. deleted  5g. Thank you for these suggestions. We decided to use a Delphi method in order to include as many people from the Equal North network as possible, which 1:1 interviews would not have enabled in the first round. The merit of open text boxes allows participants to give a wide range of focused responses. And as such we do not see this as a limitation of the study. The value of undertaking a Delphi methodology is outlined in the methods section. Combining open text responses in round 1 of the survey and responses from the workshops are outlined in the methods section. Content analysis was performed on both with a view of generating items for prioritisation. As such, we do not consider this as a limitation of the study. We have added in a line regarding the different types of experts, thank you for this.  5h. we have reworded. |
| 6. **Conclusions**   The conclusions are quite generic. More detail required to provide more direction to policy makers. For example what are the key structural issues that need to be addressed. | 7. We have added the specific question that was a topic priority from the exercise to the conclusion |
